# Supplementary material for: Foliar-feeding insects acquire microbiomes from the soil rather than the host plant
Source: Nat Commun. 2019 Mar 19;10:1254. doi: 10.1038/s41467-019-09284-w (PMC6425034; doi:10.1038/s41467-019-09284-w)
Supplement: Supplementary file 1 — Supplementary Information [file 41467_2019_9284_MOESM1_ESM.pdf]

Supplementary figures and tables accompanying paper

'Foliar-feeding insects acquire microbiomes from the soil rather than the host plant'

S. Emilia Hannula [1], Feng Zhu[1#], Robin Heinen[1,2], T. Martijn Bezemer[1,2]

[1]: *Department of Terrestrial Ecology, The Netherlands Institute of Ecology, Droevendaalsesteeg, 6708PB, Wageningen, The Netherlands*

[2]: *Institute of Biology, Section Plant Ecology and Phytochemistry, Leiden University, P.O. Box 9505, 2300 RA Leiden, The Netherlands*

# current address: Key Laboratory of Agricultural Water Resources, Hebei Key Laboratory of Soil Ecology, Center for Agricultural Resources Research, Institute of Genetic and Developmental Biology, The Chinese Academy of Sciences, Hebei, China

All authors contributed equally to this work.

Correspondence to: m.bezemer@nioo.knaw.nl

14 supplementary figures

5 supplementary tables

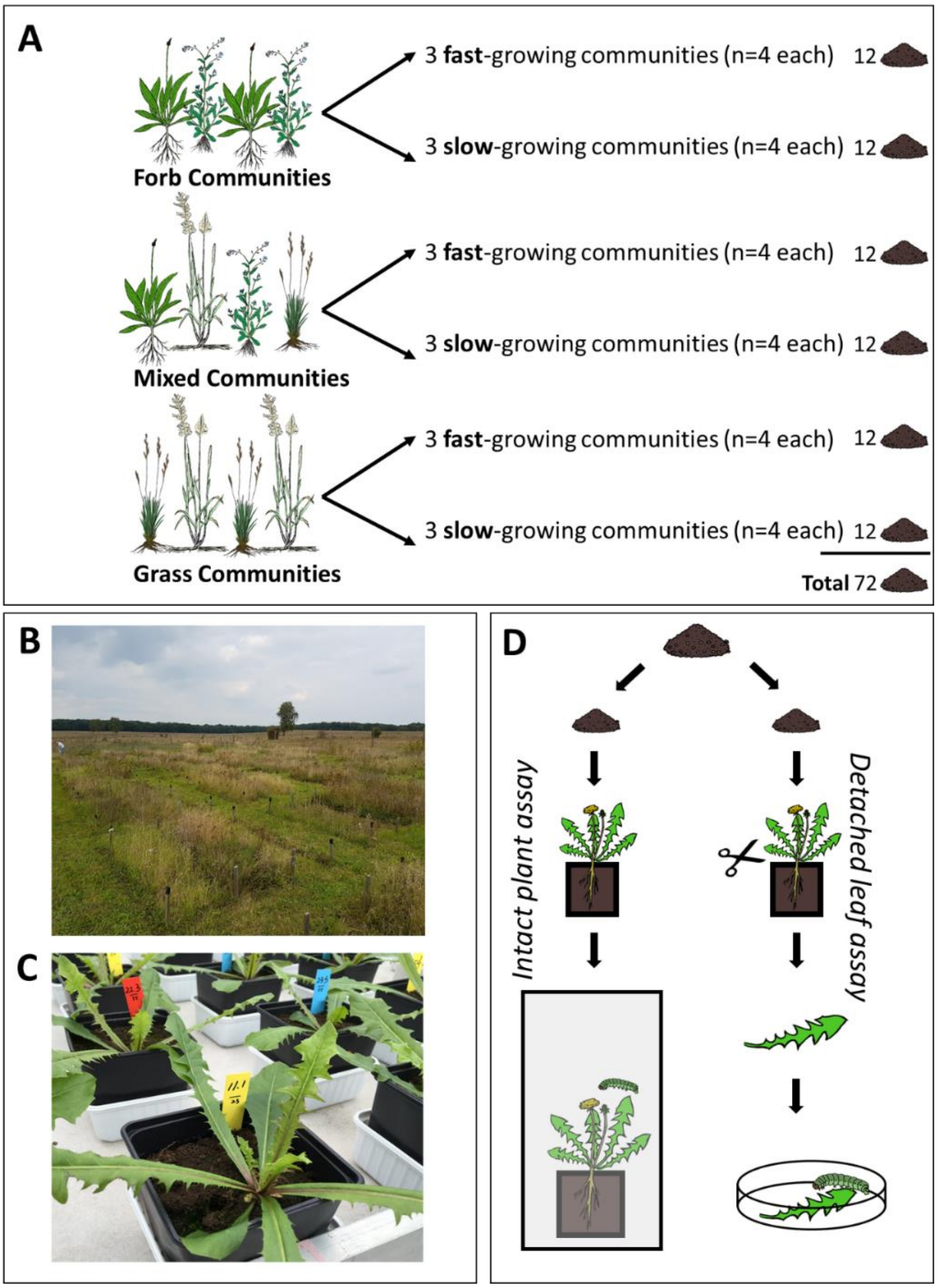

Supplementary figure 1. A. Experimental design of the field experiment from which the soils were collected. Plots sown with plant communities that consisted of only forbs, forbs and grasses, or only grasses. For each of these categories, there were three randomized slow-growing plant communities, or three randomized fast-growing plant communities (see Tables S2 and S3 for species composition). Each of the individual communities was replicated four times over four blocks in the field. **B.** Picture of the field experiment at De Mossel, Ede, The Netherlands in September 2017. **C.** *Taraxacum officinale* has a rosette growth-form but leaves generally grow upright. Except for the first few true leaves, most leaves are never in touch with the soil. **D.** Schematic overview of experimental procedure. Each donor soil was divided over two pots and one individual *T. officinale* was planted in each pot. At the onset of the caterpillar assays one plant was caged with caterpillars (intact plant assay). From the other plant, leaves were clipped and fed to caterpillars in large petri dishes (detached leaf assay).

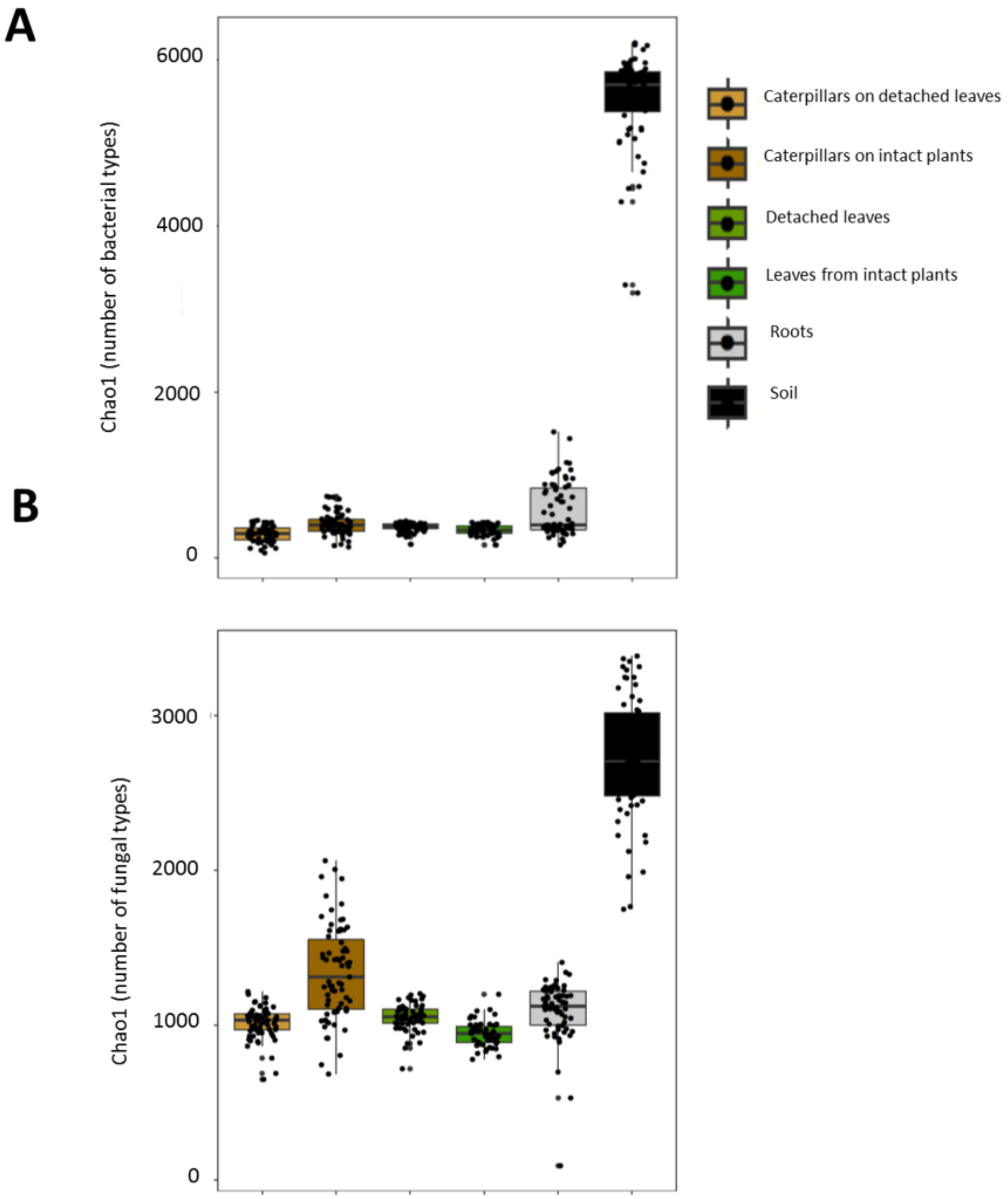

Supplementary figure 2 OTU Richness of bacteria (**A**) and fungi (**B**). The Chao1 index is shown for caterpillars on intact plants (dark brown), caterpillars on detached leaves (light brown), leaves from plants from the intact-plant assay (dark green) and leaves from plants from the detached-leaf assay (light green), roots (grey) and soil (black). The Tukey box-and-whisker plots depict median number of phyla and classes in each compartment and variation is shown in the scatter.

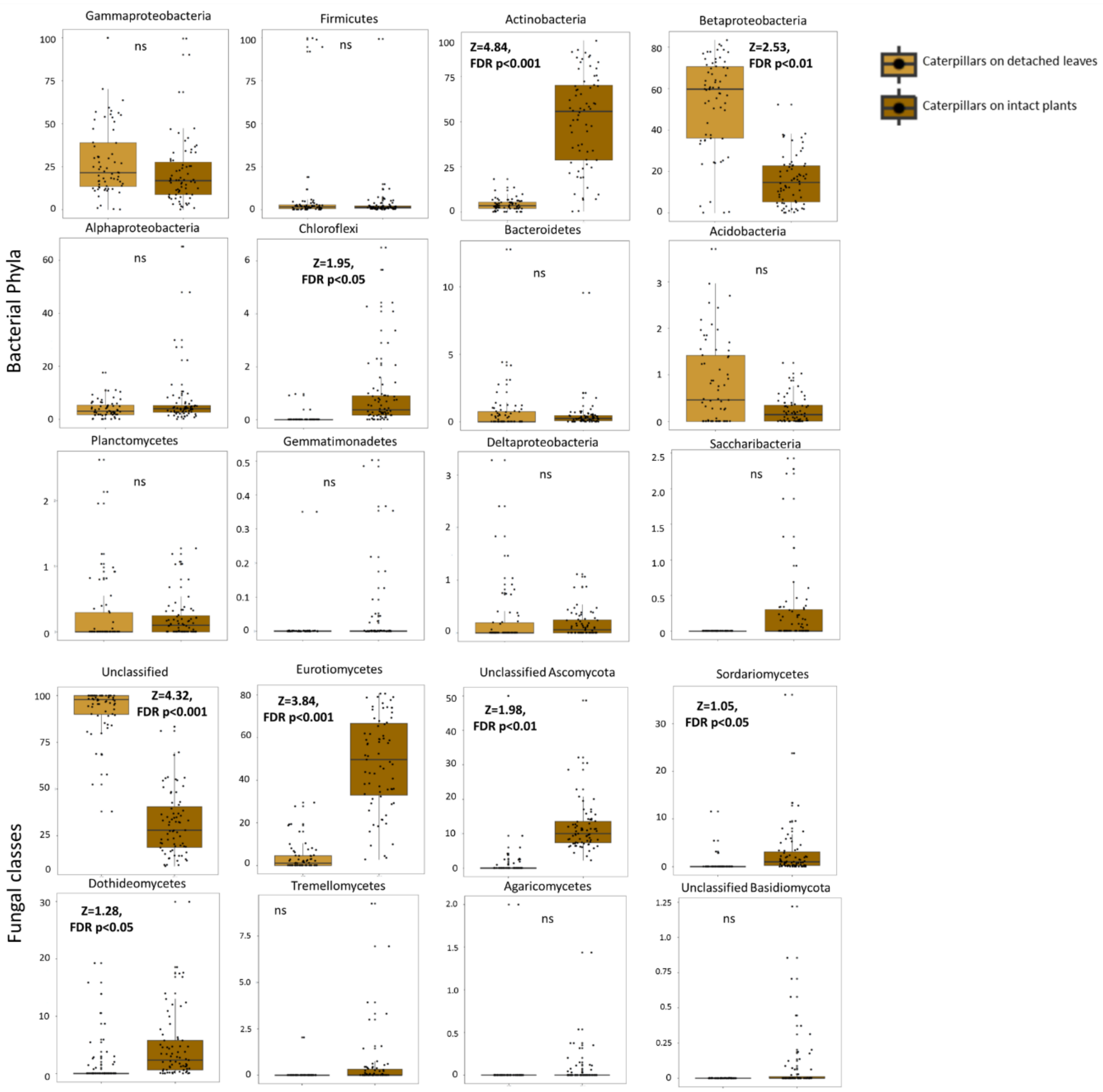

Supplementary figure 3 Relative abundance of bacterial phyla and fungal classes inside caterpillars kept on intact plants (dark brown) and caterpillars fed detached leaves (light brown). The Tukey box-and-whisker plots depict median relative abundance of phyla and classes in caterpillars on detached leaves and on intact plants and variation is shown in the scatter. The plots are organized by abundance, in decreasing order. The z-values derived from a GLM model and the FDR corrected p-values for bacterial phyla and fungal classes that significantly differ between the caterpillars on intact plants and on detached leaves are presented in the panels.

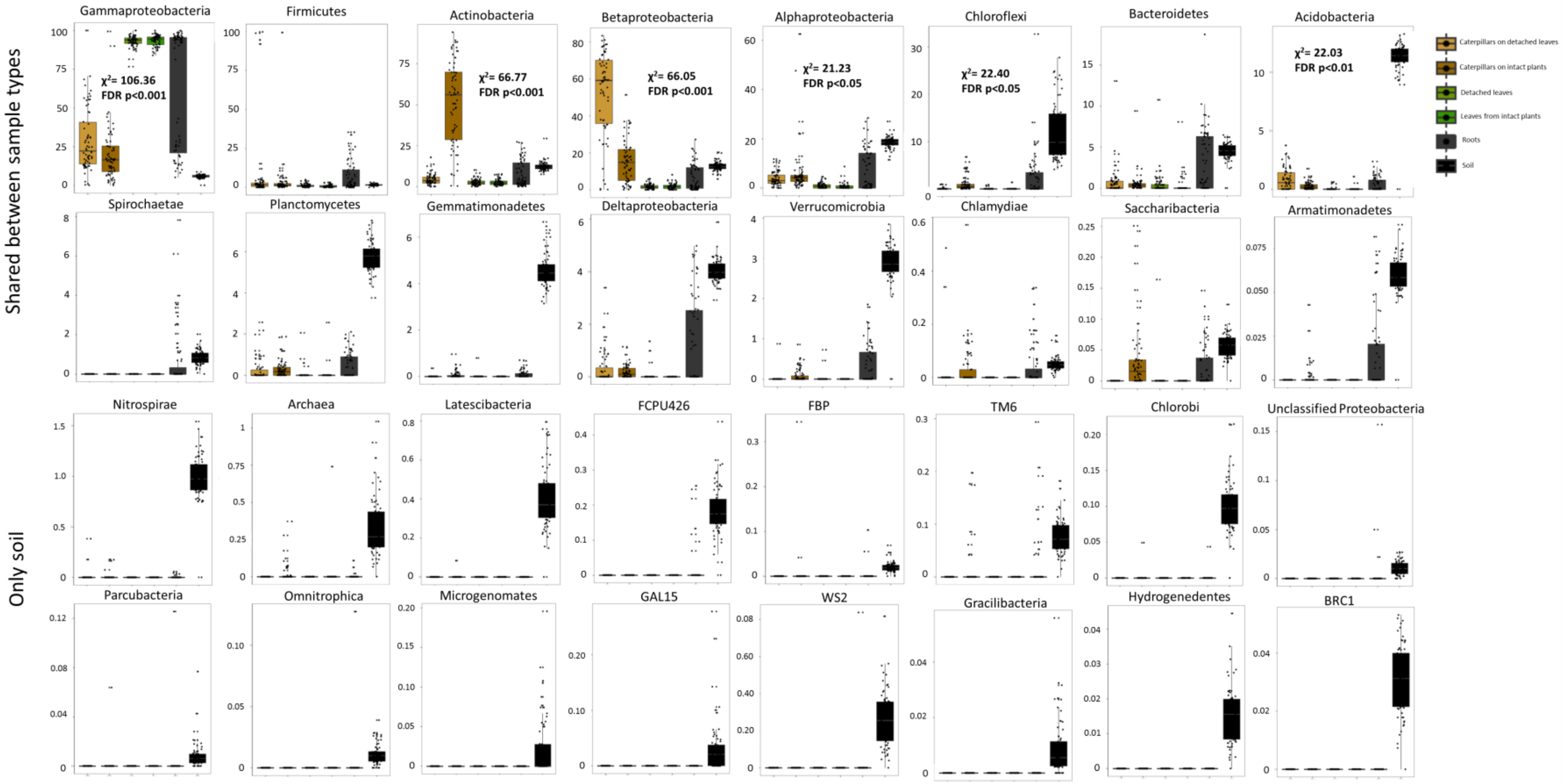

Supplementary figure 4 Relative abundance of bacterial phyla in caterpillars, leaves, roots and soil. The upper 16 panels represent phyla shared between multiple sample types and the lower 16 panels are rare in other environments than soil (Fig. 1A). The light brown color represents microbes in caterpillars fed on detached leaves, dark brown represents microbes in caterpillars kept on intact plants, light green represents microbes in leaves from plants of the detached-leaf assay plants; dark green represents leaves from plants from the intact-plant assay, grey represents microbes inside the roots, and black represents microbes in the soil samples. The Tukey box-and-whisker plots depict median relative abundance of each phyla and variation is shown in the scatter. The phyla are ordered based on their relative abundance from highest to lowest. Significant FDR corrected p-values and chisquare test values of the GLM model are presented if significant in the panels.

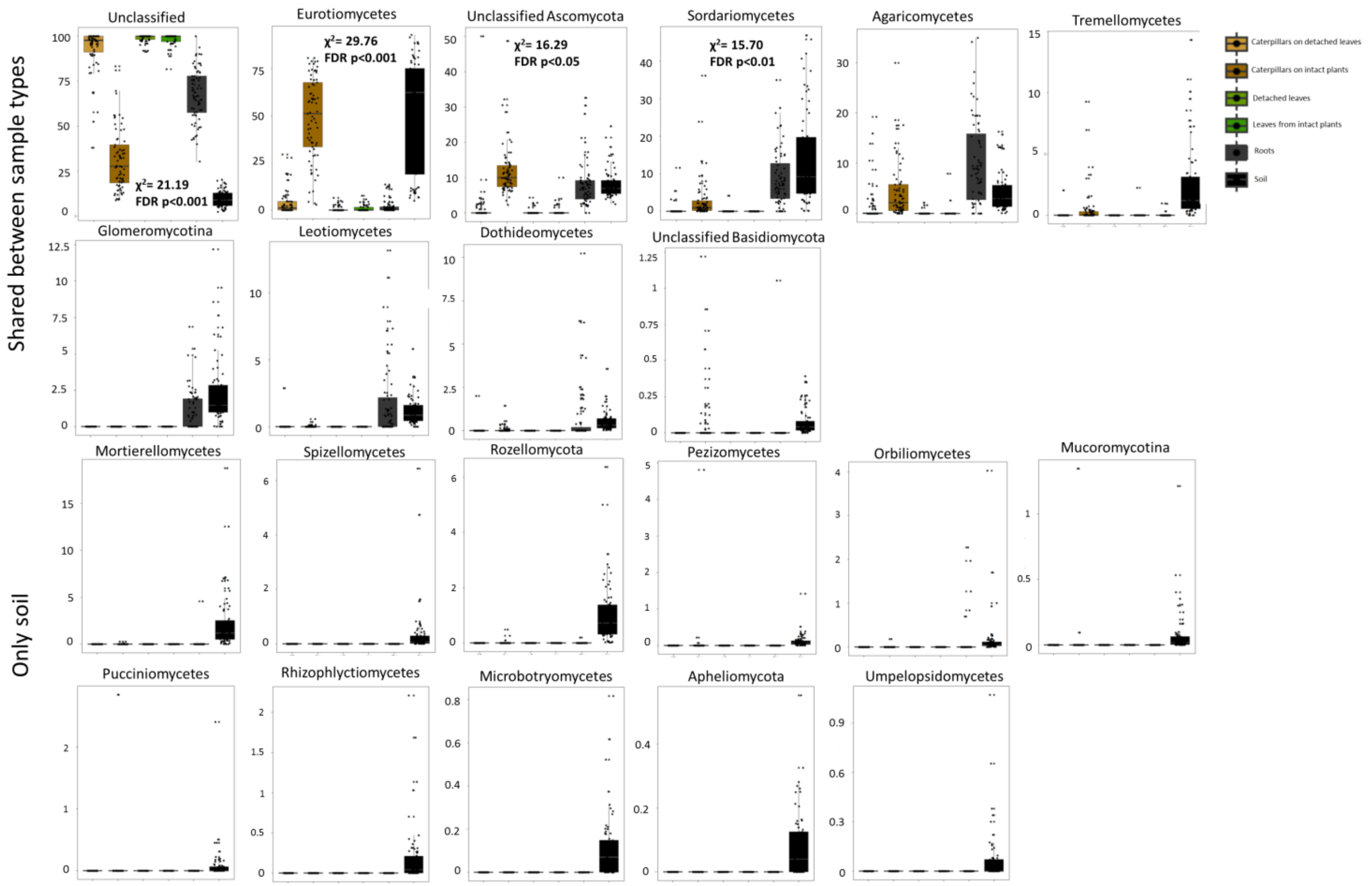

Supplementary figure 5 Relative abundance of fungal classes in caterpillars, leaves, roots and soil. The first 10 panels are shared between multiple sample types and the last 11 are rare in other environments than soil (Fig. 1B). Bars with a light brown color represent microbes in caterpillars fed on detached leaves, dark brown represents microbes in caterpillars kept on intact plants, light green represents microbes in leaves from plants of the detached-leaf assay plants; dark green represents leaves from plants from the intact-plant assay, grey represents microbes inside the roots, and black represents microbes in the soil samples. The Tukey box-and-whisker plots depict median relative abundance of each class and variation is shown in the scatter. The classes are ordered based on their relative abundance from highest to lowest. Significant FDR corrected p-values and chisquare test values of the GLM model are presented if significant in the panels

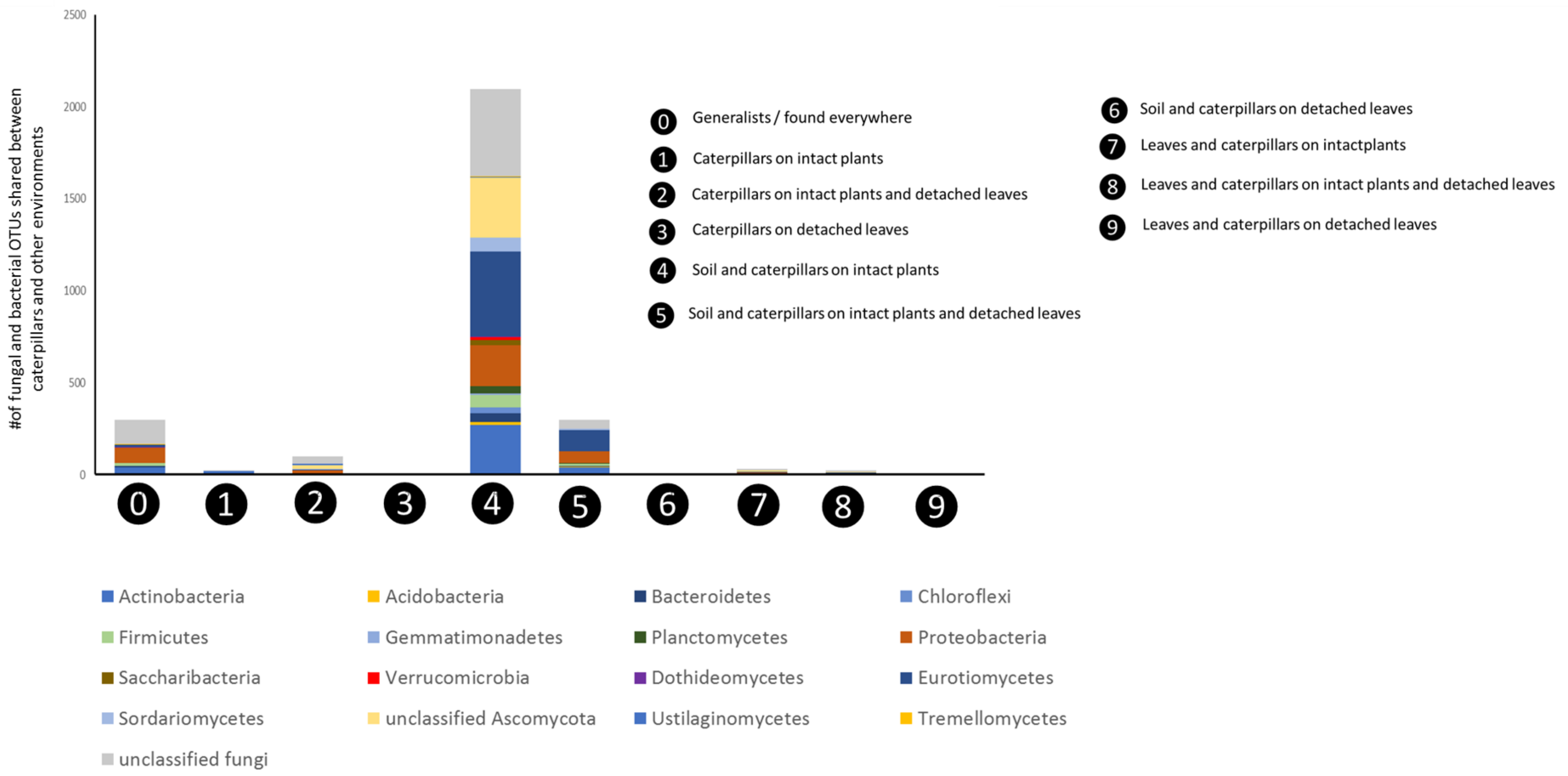

Supplementary figure 6 The identity and the number of the OTUs shared between the environments (0-9) depicted in Fig. 2C. Only phyla and classes with more than 5 OTUs present are presented in the figure.

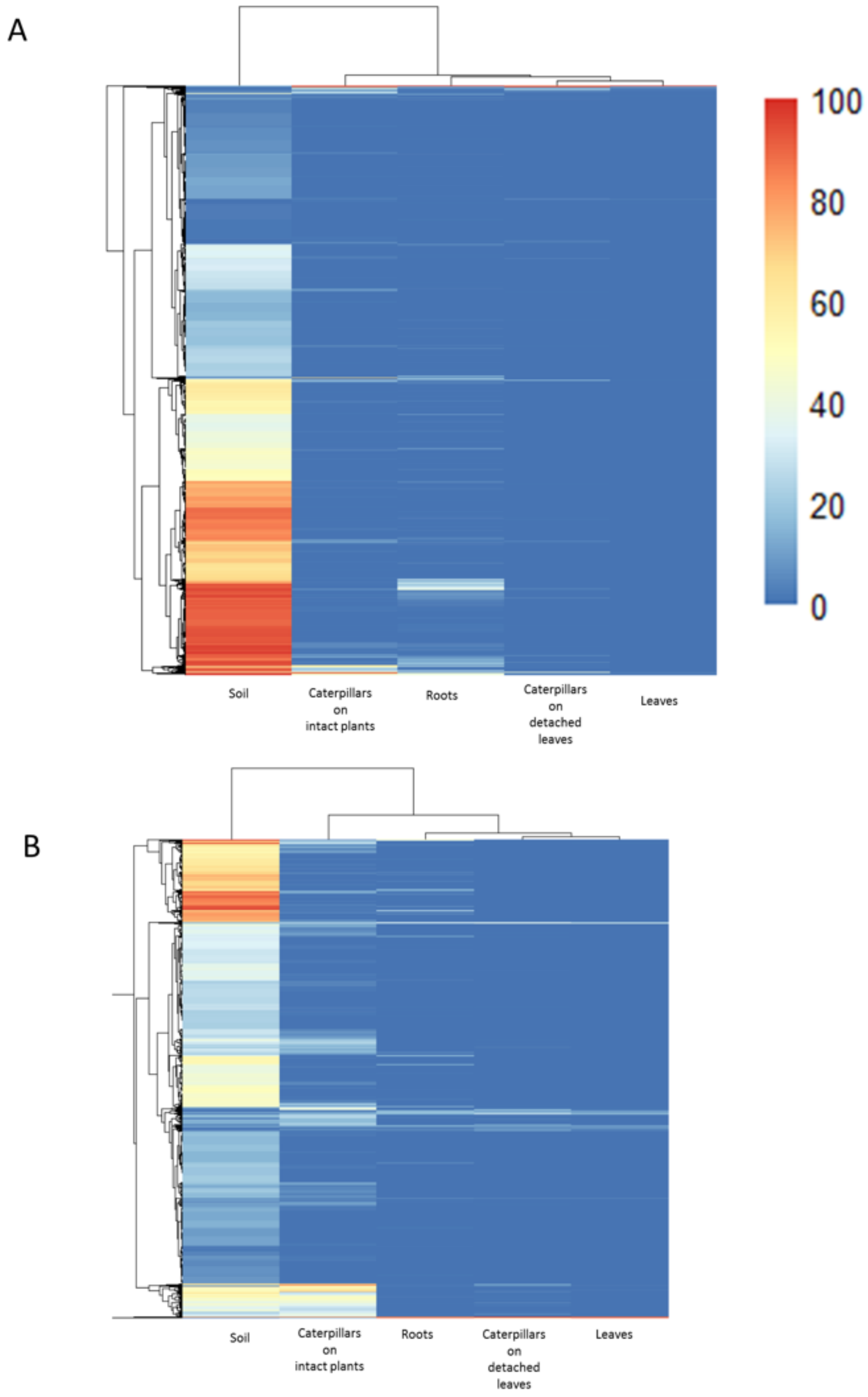

Supplementary figure 7 Heat maps showing all bacterial (**A**) and fungal (**B**) OTUs with average abundance of more than <0.1% presence in samples (as % of samples present) in different compartments (soil, caterpillars on intact plants, caterpillars on detached leaves, roots and leaves), and how compartments cluster with each other. The red color indicates that a class is found in 100% of the samples while blue colors indicate that it is found in 0-30% of samples.

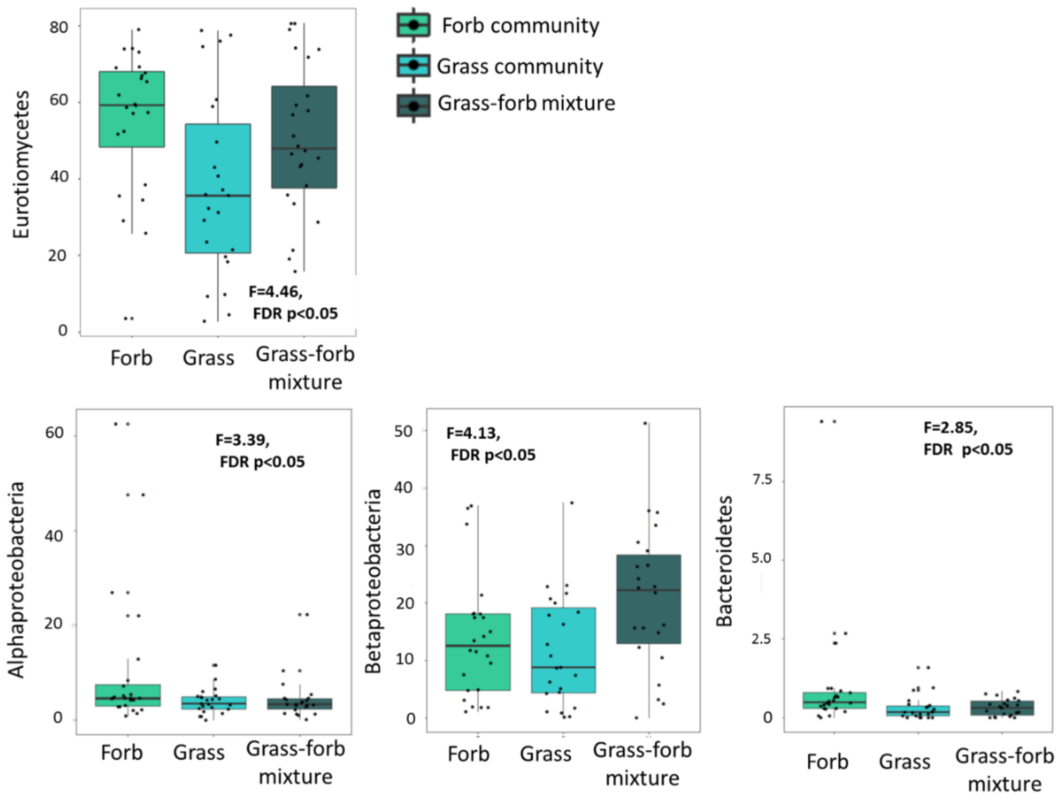

Supplementary figure 8 Fungal classes and bacterial phyla in caterpillars kept on intact plants that are significantly affected by the plant community that previously grew in the soil, as presented in Fig. 3B&F. Relative abundances are depicted and they are presented in order of abundance. The Tukey box-and-whisker plots depict median relative abundance of phyla and classes and variation is shown in the scatter. Statistical results of LME on the relative abundances are also presented. Light green represents soil from grass communities, turquoise represents soil from forb communities and dark green represents soil from mixed communities.

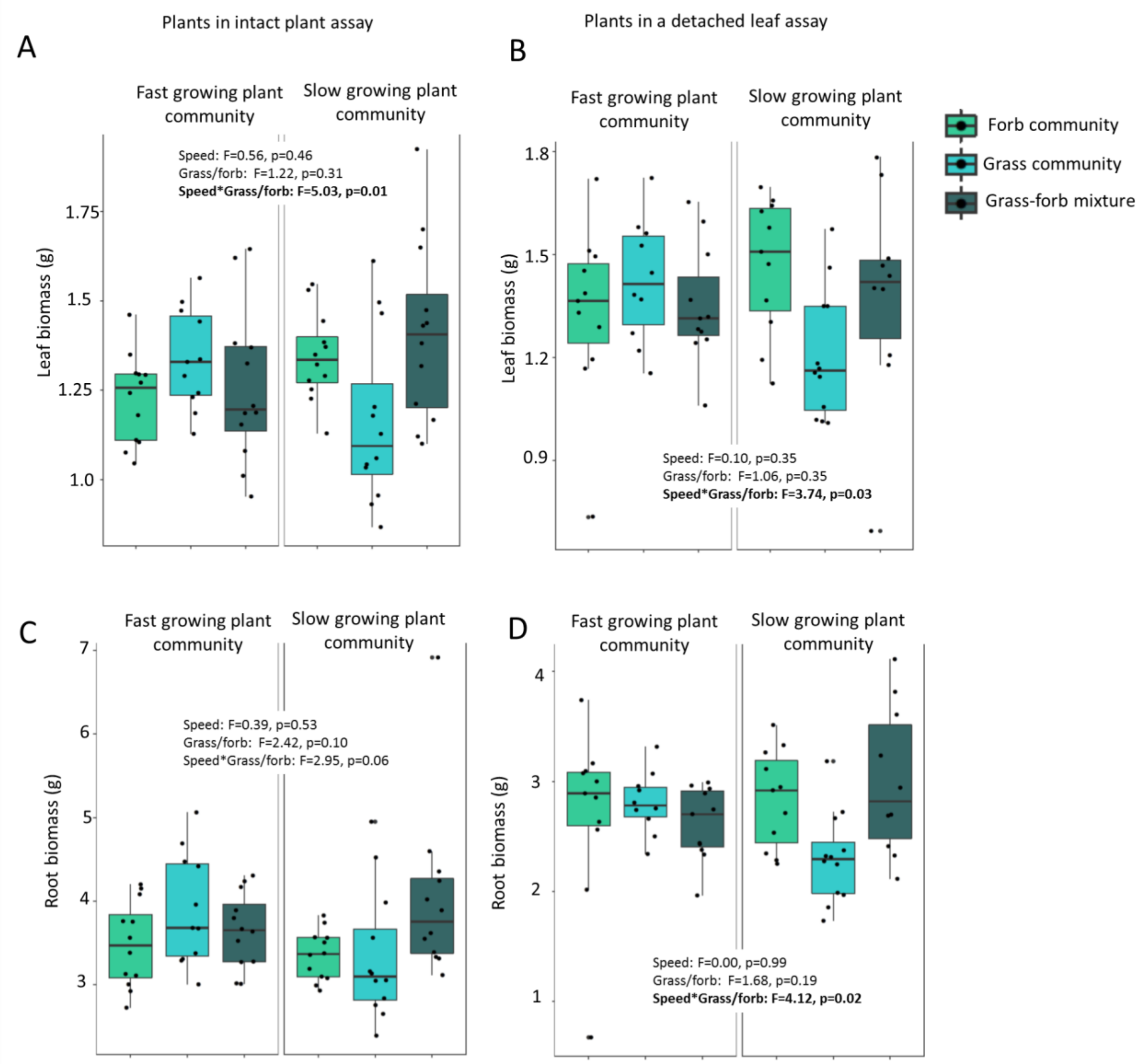

Supplementary figure 9 Average leaf (**A & B**) and root (**C & D**) biomass of dandelion plants from the assay with intact plants with caterpillars (**A & C**) and from the assay with detached leaves (**B & D**) grown in soils with a legacy of fast or slow growing plants, and a legacy of forb, grass or mixed plant communities. The Tukey box plots depict median biomass of dandelion in different legacies and variation is shown in the scatter. Light green represents soil from grass communities, turquoise represents soil from forb communities and dark green represents soil from mixed communities. F-values and P-values from a LME are presented in panels and significant p-values are marked in bold.

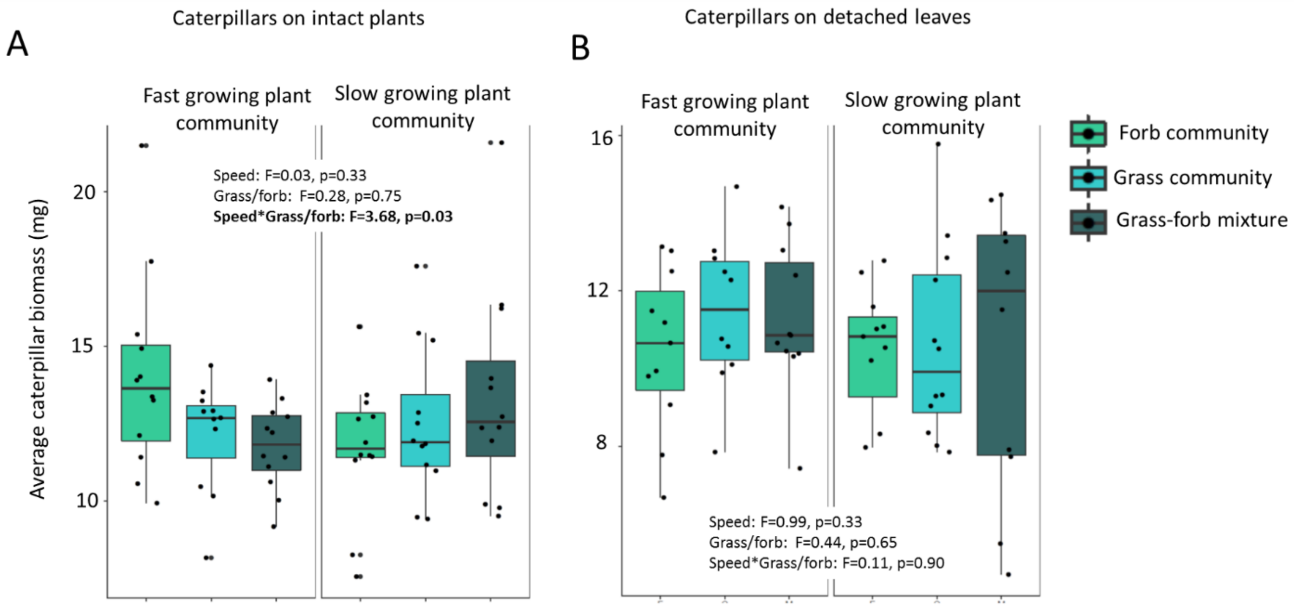

Supplementary figure 10 Average caterpillar biomass on intact plants (**A**) and on detached leaves (**B**) from plants grown in soils with a legacy of fast or slow growing plants, and a legacy of forb, grass or mixed plant communities. The Tukey box plots depict median biomass of caterpillars in different plant legacies and variation is shown in the scatter. Light green represents soil from grass communities, turquoise represents soil from forb communities and dark green represents soil from mixed communities. F-values and p-values from a LME are presented and significant p-values are marked in bold.

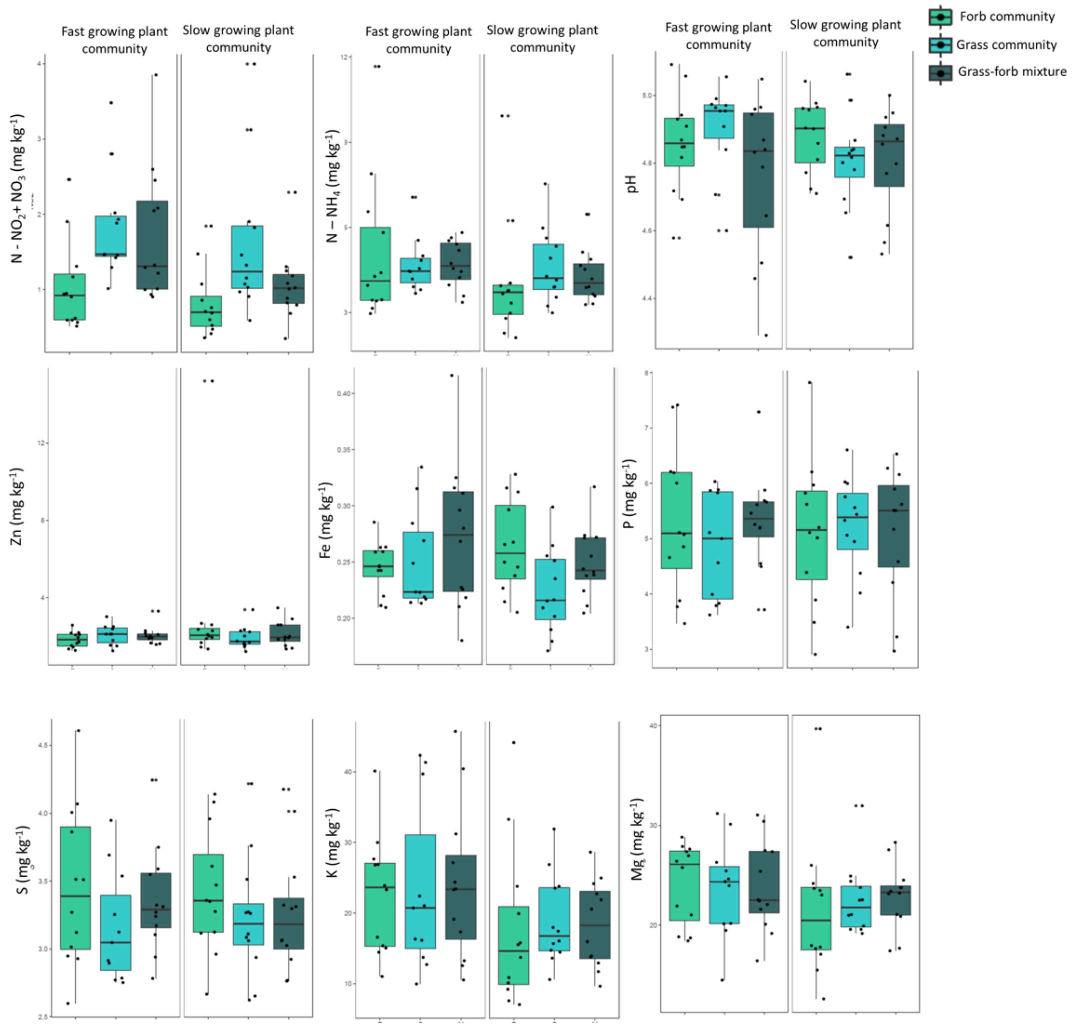

Supplementary figure 11. Chemical composition of soils with a legacy of fast or slow growing plants, and a legacy of forb, grass or mixed plant communities. The Tukey box-and-whisker plots depict median measurement of chemistry in soils with different plant legacies and variation is shown in the scatter. Light green represents soil from grass communities, turquoise represents soil from forb communities and dark green represents soil from mixed communities. The results from a LME are presented in supplementary table 1.

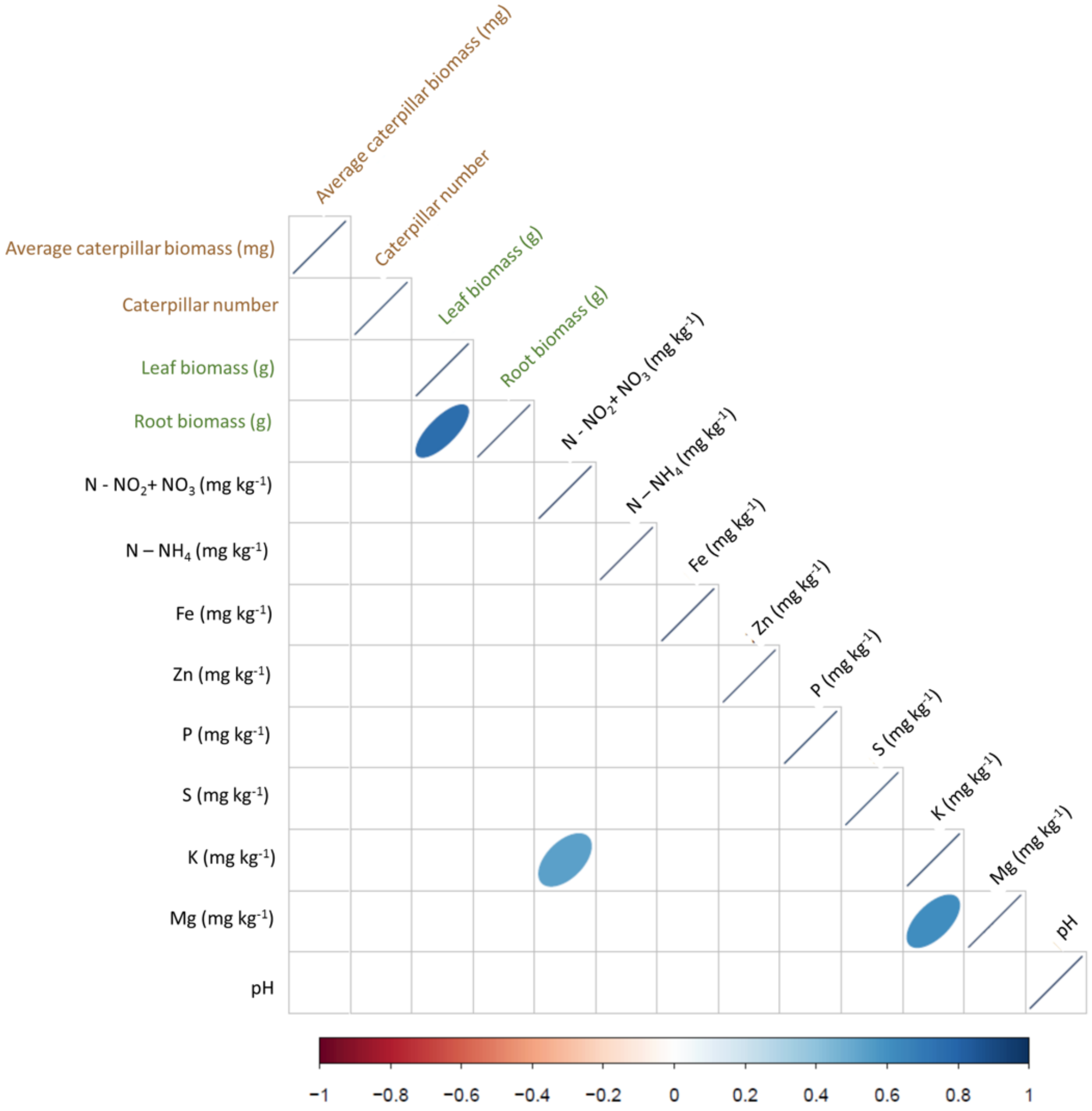

Supplementary figure 12 Correlation matrix for soil chemistry variables and caterpillar and plant performance. Correlations are based on Pearson correlation coefficients. Average caterpillar biomass (brown), caterpillar survival (brown) per plant, and leaf- and root biomass (green) per soil sample was used. The scale color of the filled squares indicates the strength of the correlation (r) and whether it is negative (red) or positive (blue). All correlations are corrected for FDR and only significant correlations with $p<0.05$ are shown. If the correlation is not significant, the box is left white.

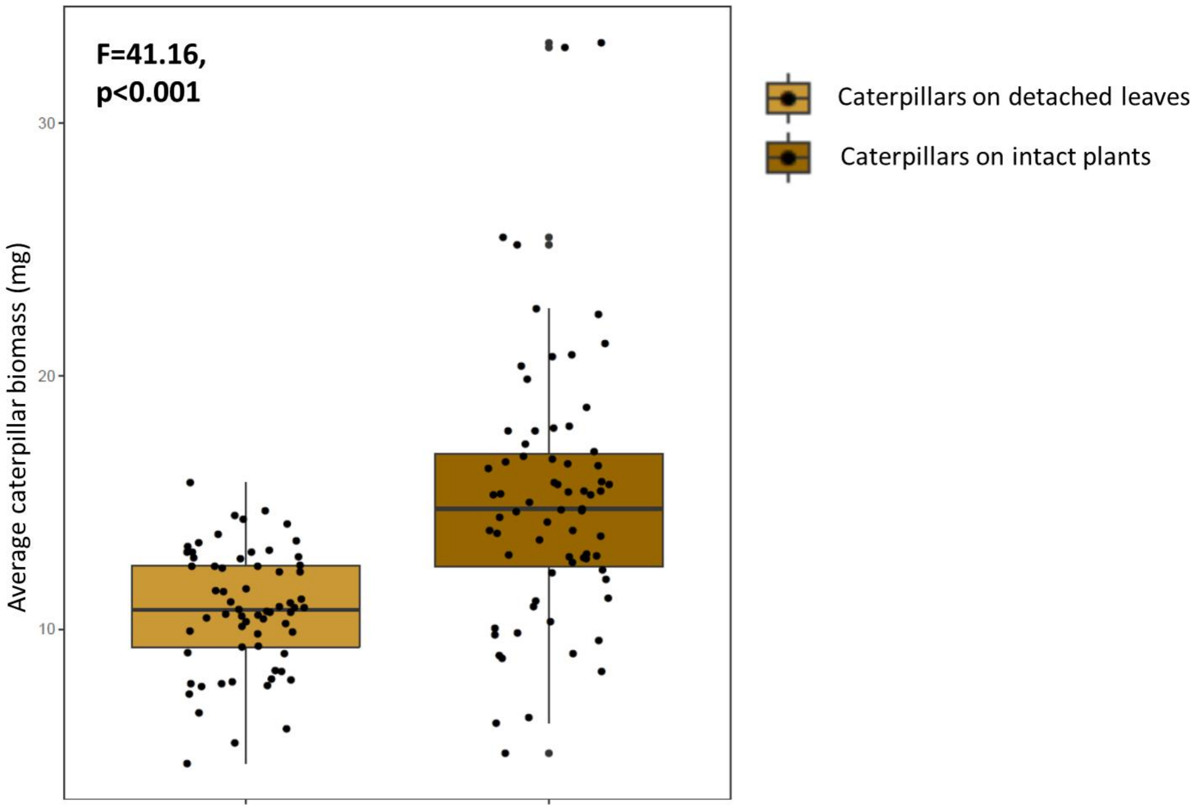

Supplementary figure 13 Tukey box-and-whisker plot showing median caterpillar biomass after feeding on whole plants for 14 days (dark brown) or detached leaves for 5 days (light brown). The F-value and p-value of a LME are also presented.

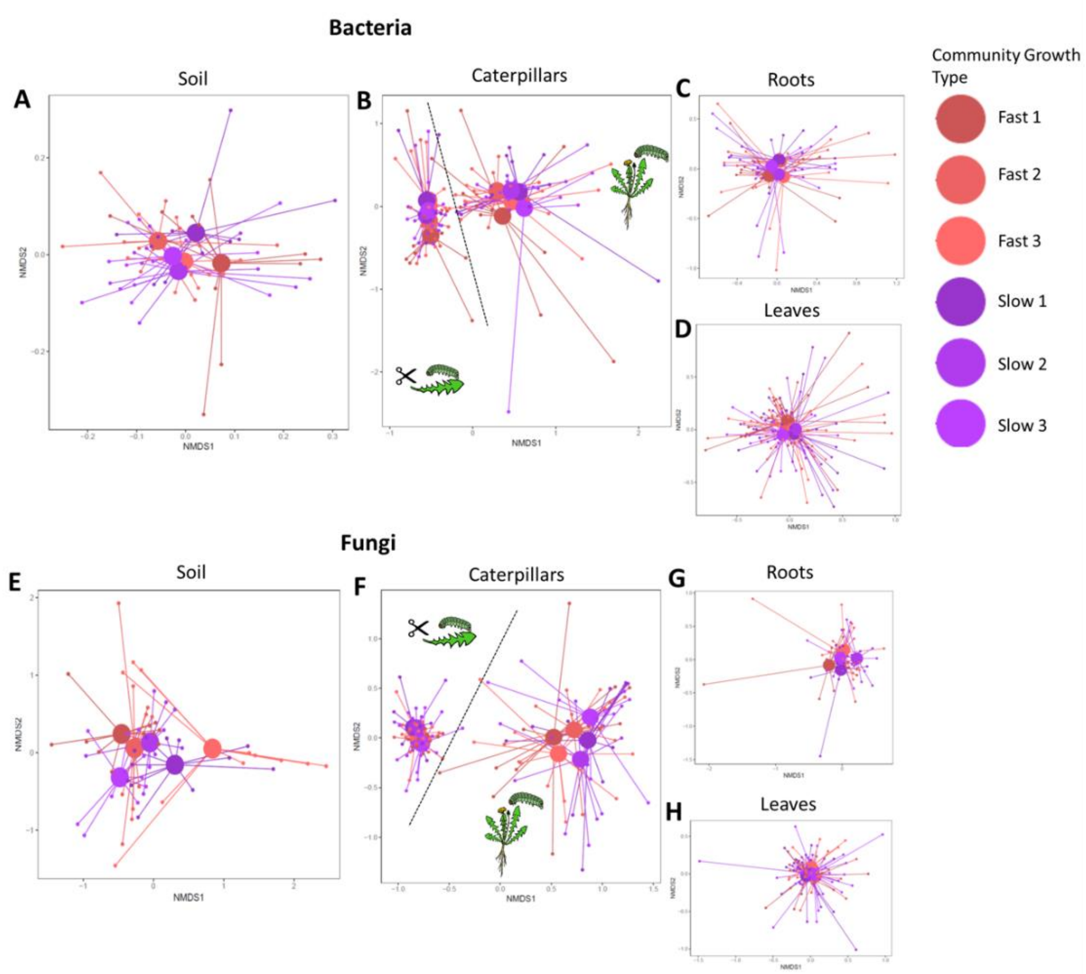

Supplementary figure 14 Effects of plant community growth rate (fast or slow) on the community composition of bacteria (A-D) and fungi (E-H) in caterpillars, leaves, roots and soil. NMDS plots are based on Bray-Curtis similarity. The 2D stress value for each panel ranges between 0.11-0.18. **A-F** microbiomes originating from soils conditioned by fast growing species are represented by markers in shades of red and microbiomes originating from soils conditioned by slow growing species are represented by markers in shades of blue. The centroids are marked with larger markers; smaller markers depict individual samples. **A&E** show the effect on soil microbiomes, **B&F** on microbiomes in caterpillars both on intact plants and on detached leaves, **C&G** on root microbiomes, and **D&H** on leaf microbiomes.

Supplementary table 1 Effect of plant **community** type (communities with grasses only, with forbs only, and with mixtures of grasses and forbs), **growth rate** (fast- and slow-growing plant communities), and their **interaction (C x GR)** on soil chemistry during the conditioning phase in the field. Mean values are presented in Supplementary Fig. 11. Effects that were significant after correction for FDR are marked in bold.

	**Community** (grass/forb/mixture)	**Growth rate** (fast/slow)	**C x GR**
	F (p)	F (p)	F (p)
pH	1.9 (0.153)	0.0 (0.848)	1.3 (0.285)
NO_2+NO_3	**7.2 (0.002)**	4.8 (0.312)	0.7 (0.481)
NH_4	0.1 (0.950)	1.8 (0.189)	0.5 (0.597)
Fe	2.0 (0.141)	1.1 (0.297)	2.1 (0.132)
Zn	0.6 (0.562)	1.1 (0.289)	1.3 (0.274)
P	0.2 (0.804)	0.0 (0.914)	0.5 (0.626)
S	1.7 (0.197)	0.0 (0.897)	0.2 (0.836)
K	0.1 (0.097)	5.1 (0.059)	0.0 (0.024)
Mg	0.1 (0.930)	2.0 (0.167)	0.2 (0.802)

Supplementary table 2 List of plant species sown in the field plots.

Fast-growing grasses	Slow-growing grasses	Fast-growing forbs	Slow-growing forbs
Dactylis glomerata (Dg)	*Arrhenaterum elatius* (Ae)	*Plantago lanceolata* (Pl)	*Tripleurospermum maritimum* (Tm)
Holcus lanatus (Hl)	*Briza media* (Bm)	*Rumex acetosella* (Ra)	*Clinopodium vulgare* (Cv)
Alopecurus pratensis (Ap)	*Trisetum flavescens* (Tf)	*Achillea millefolium* (Am)	*Geranium molle* (Gem)
Agrostis capillaris (Ac)	*Anthoxanthum odoratum* (Ao)	*Taraxacum officinale* (To)	*Myosotis arvensis* (Ma)
Lolium perenne (Lp)	*Deschamptia flexuosa* (Df)	*Epilobium hirsutum* (Eh)	*Galium mollugo* (Gam)
Phleum pretense (Pp)	*Festuca ovina* (Fo)	*Crepis capillaris* (Cc)	*Gnaphalium sylvaticum* (Gs)

Supplementary table 3 Composition of the sown grass, forb and mixed communities consisting of fast and slow growing plants. Species abbreviations are explained in Supplementary table 2.

Type	Community	Grasses			Forbs		
Fast-growing grasses	1	Dg	Hl	Ap			
	2	Ac	Lp	Hl			
	3	Pp	Dg	Lp			
Fast-growing forbs	4				Pl	Cc	Ta
	5				Ra	Cc	Am
	6				Am	Eh	To
Fast-growing mixtures	7	Dg	Hl	Ap	Pl	Cc	Ta
	8	Ac	Lp	Hl	Ra	Cc	Am
	9	Pp	Dg	Lp	Am	Eh	To
Slow-growing grasses	10	Ae	Bm	Fo			
	11	Bm	Tf	Ao			
	12	Ao	Df	Tf			
Slow-growing forbs	13				Tm	Cv	Gem
	14				Cv	Gs	Ma
	15				Tm	Ma	Gam
Slow-growing mixtures	16	Ae	Bm	Fo	Tm	Cv	Gem
	17	Bm	Tf	Ao	Cv	Gs	Ma
	18	Ao	Df	Tf	Tm	Ma	Gam

Supplementary table 4 Recipe for the artificial diet that was used to feed *Mamestra brassicae* in the first larval stage.

**Ingredients**
5L water
140g agar
800g corn flour
250g beer yeast
150g wheat germs
10g sorbic acid
40g ascorbic acid
8g nipagin (methyl-4-hydroxybenzoate)
0.5g streptomycin
**Preparation**
Bring 4L water to a boil, while dissolving the agar in 1L cold water. When boiling, turn down the heat and add corn flour, yeast and wheat germs and stir until homogenized. Add sorbic acid and nipagin until homogenized. Add ascorbic acid and streptomycin and stir until homogenized. Freeze in small portions and thaw before use for rearing.

Supplementary table 5 Number of samples left in each compartment after filtering the samples with too few or too many reads.

	Compartment	Fungi (n=72)	Bacteria (n=72)
Intact plant assay	Caterpillars	71	68
	Leaves	62	65
	Roots	67	70
	Soil	65	68
Detached leaf assay	Caterpillars	68	69
	Leaves	64	70
