# Supplementary material for: Foliar-feeding insects acquire microbiomes from the soil rather than the host plant
Source: Nat Commun. 2019 Mar 19;10:1254. doi: 10.1038/s41467-019-09284-w (PMC6425034; doi:10.1038/s41467-019-09284-w)
Supplement: Supplementary file 3 — Reporting Summary [file 41467_2019_9284_MOESM3_ESM.pdf]

nature research

Corresponding author(s): T.M. Bezemer

Reporting Summary

Nature Research wishes to improve the reproducibility of the work that we publish. This form provides structure for consistency and transparency in reporting. For further information on Nature Research policies, see Authors & Referees and the Editorial Policy Checklist.

Statistical parameters

When statistical analyses are reported, confirm that the following items are present in the relevant location (e.g. figure legend, table legend, main text, or Methods section).

n/a	Confirmed	
☐	☒	The exact sample size (*n*) for each experimental group/condition, given as a discrete number and unit of measurement
☐	☒	An indication of whether measurements were taken from distinct samples or whether the same sample was measured repeatedly
☐	☒	The statistical test(s) used AND whether they are one- or two-sided<br>*Only common tests should be described solely by name; describe more complex techniques in the Methods section.*
☐	☒	A description of all covariates tested
☐	☒	A description of any assumptions or corrections, such as tests of normality and adjustment for multiple comparisons
☐	☒	A full description of the statistics including central tendency (e.g. means) or other basic estimates (e.g. regression coefficient) AND variation (e.g. standard deviation) or associated estimates of uncertainty (e.g. confidence intervals)
☐	☒	For null hypothesis testing, the test statistic (e.g. *F*, *t*, *r*) with confidence intervals, effect sizes, degrees of freedom and *P* value noted<br>*Give P values as exact values whenever suitable.*
☐	☒	For Bayesian analysis, information on the choice of priors and Markov chain Monte Carlo settings
☐	☒	For hierarchical and complex designs, identification of the appropriate level for tests and full reporting of outcomes
☐	☒	Estimates of effect sizes (e.g. Cohen's *d*, Pearson's *r*), indicating how they were calculated
☐	☒	Clearly defined error bars<br>*State explicitly what error bars represent (e.g. SD, SE, CI)*

Our web collection on statistics for biologists may be useful.

Software and code

Policy information about availability of computer code

Data collection	pipelines used for bioinformatics<br>Bacteria: De Hollander, M. nioo-knaw/hydra: 1.3.3 (https://doi.org/10.5281/zenodo.884028), 2017).<br>Fungi: https://github.com/hsgweon/pipits
Data analysis	R code is available upon request, we used standard approaches and packages such as Phyloseq, Vegan

For manuscripts utilizing custom algorithms or software that are central to the research but not yet described in published literature, software must be made available to editors/reviewers upon request. We strongly encourage code deposition in a community repository (e.g. GitHub). See the Nature Research guidelines for submitting code & software for further information.

Data

Policy information about availability of data

All manuscripts must include a data availability statement. This statement should provide the following information, where applicable:

- Accession codes, unique identifiers, or web links for publicly available datasets
- A list of figures that have associated raw data
- A description of any restrictions on data availability

Paired-end DNA sequencing reads for this project have been deposited in the European Nucleotide Archive under accession number PRJEB27512 and all other data in Dryad under https://doi.org/10.5061/dryad.99504fd

Field-specific reporting

Please select the best fit for your research. If you are not sure, read the appropriate sections before making your selection.

☐ Life sciences ☐ Behavioural & social sciences ☒ Ecological, evolutionary & environmental sciences

For a reference copy of the document with all sections, see nature.com/authors/policies/ReportingSummary-flat.pdf

Ecological, evolutionary & environmental sciences study design

All studies must disclose on these points even when the disclosure is negative.

Study description	We sequenced microbiomes of soils, roots, leaves and aboveground insects. Soils originated from 18 different plant communities growing in four blocks in a field experiment, totalling 72 plant communities. Dandelion was grown in all soils in twofold resulting in 144 pots in a greenhouse experiment. One of the two plants on each soil was used to collect leaves that were fed to caterpillars in petri-dishes, while the other plant was caged and other caterpillars were introduced in these cages.
Research sample	Test plant: clonal line of Taraxacum officinale.<br>Caterpillar: Mamestra brassicae (fed on artificial diet until they reached second larval instar)<br>Soil: collected from a field experiment in a natural grassland, De Mossel, Ede, The Netherlands<br>Bacterial DNA was analyzed based on 16S<br>Fungal DNA was analyzed based on ITS-2 region
Sampling strategy	Sampling of the soils in the field consisted of 20 individual soil cores (2 cm diameter, 10 cm depth) in each plot (83 x 250 cm). Cores were homogenized before use in the greenhouse experiment. Soil samples of the greenhouse pots were taken from the intact plants at the moment of harvest, by removing bulk soil by shaking and collecting the soil that tightly attached to the roots. Soil samples were stored at -80 degrees C. Leaf samples were taken using a 25mm leaf puncher (3 leaf punches from 3 individual leaves per plant, combined into one sample) and flash frozen in liquid nitrogen and stored at -80 degrees C. Root samples were washed on a sieve (350 µm) and fine roots were selected. Roots were then sonicated in a BRANSONIC ultrasonic cleaner (Bransonic ultrasonics, Danbury, USA) for 10 min (ten cycles of 30s ultrasonic burst, followed by 30s rest) in order to disrupt microbes that were attached to the root exterior. Samples were then rinsed with sterilized water and frozen at -80 degrees C. Caterpillars were collected using forceps and were surface sterilized using 2.5% bleach and then rinsed with sterilized water. Caterpillar samples were then sonicated in a BRANSONIC ultrasonic cleaner (Bransonic ultrasonics, Danbury, USA) for 10 min (ten cycles of 30s ultrasonic burst, followed by 30s rest) in order to disrupt microbes that were attached to the caterpillar cuticle. Samples were then rinsed with sterilized water and then frozen at -80 degrees C. No sample-size calculation was performed. Sample-sizes followed the experimental design of an established field experiment with four blocks, including replication at different levels within treatments.
Data collection	Caterpillar, plant leaf and root samples were lyophilized prior to DNA extractions. Bead beating and DNA extraction were performed with the MP Biomedical FastDNA™ Spin Kit. For the soil samples, DNA was extracted using Qiagen DNeasy PowerSoil Kit. Sequencing data was generated in McGill genome Quebec and processed using above mentioned pipelines. Raw data in fastQC format is available in the European Nucleotid Archive (ENA)
Timing and spatial scale	Soil was collected from the experimental field on 02-02-2017. The plants were grown for five weeks. Then the plants of the detached-leaf assay were used for 5 days. On intact plants, caterpillars were kept for 14 days. At seven weeks all plants were harvested.
Data exclusions	From sequencing data; samples that had read numbers that were less than 1/3 or three times higher than the mean read number of that sample type, were considered outliers and removed from analysis. There were four replicates and with exclusions in all treatments there were at least 3 replicates left. Number of samples analyzed for bacterial and fungal communities (out of a total of 72 samples): Caterpillars on caged plants: Bacteria=68, Fungi=71; Caterpillars on detached leaves: Bacteria=69, Fungi=68; Leaves from caged plants: Bacteria=65, Fungi=62; Leaves from plants used for detached leaves: Bacteria=70, Fungi=64; Roots: Bacteria=70, Fungi=67; Soil: Bacteria=68, Fungi=65.
Reproducibility	To compare between sequencing runs, a mock community of known composition was used as internal control. We used standard commercial kits and open source pipelines for bioinformatic analysis. All statistical analyses were performed in R. Codes are available upon request or can be uploaded as supplementary information if needed.
Randomization	The soil samples were taken from a field study that had a randomized block design with each block containing all treatments. The

Randomization	greenhouse experiment contained the same replication (18 soil types from different communities x 4 replicates x 2 insect treatments = 144 pots). Pots were randomized in the greenhouse upon planting at the start of the study.
Blinding	Numbered codes were used for sample handling to ensure blind handling.

Did the study involve field work? ☒ Yes ☐ No

Field work, collection and transport

Field conditions	Soil was collected from a field experiment in a grassland in a temperate climate, 800 mm rainfall per year, monthly mean temperature 3 to 18 C
Location	De Mossel, Ede, The Netherlands, 52o04' N, 05o44' E
Access and import/export	The area was freely accessible with permission from Natuurmonumenten who manage the area
Disturbance	Existing vegetation was removed in 2015 two years prior to the experiment.

Reporting for specific materials, systems and methods

Materials & experimental systems

n/a	Involved in the study	
☒	☐	Unique biological materials
☒	☐	Antibodies
☒	☐	Eukaryotic cell lines
☒	☐	Palaeontology
☐	☒	Animals and other organisms
☒	☐	Human research participants

Methods

n/a	Involved in the study	
☒	☐	ChIP-seq
☒	☐	Flow cytometry
☒	☐	MRI-based neuroimaging

Animals and other organisms

Policy information about studies involving animals; ARRIVE guidelines recommended for reporting animal research

Laboratory animals	caterpillars were reared on artifical diet
Wild animals	no wild animals
Field-collected samples	no animals were collected in the field
